# Case report: Kidney perivascular epithelioid cell tumor treated with anti-VEGFR tyrosine kinase inhibitor and MTOR inhibitor

**DOI:** 10.3389/fonc.2022.966818

**Published:** 2022-11-16

**Authors:** Ninggang Zhang, Yaqiong Ren, Likun Zan, Xuting Zhang, Jian Zhao, Lu Wen, Yusheng Wang

**Affiliations:** ^1^ Department of Gastrointestinal Oncology, Shanxi Province Cancer Hospital/Shanxi Hospital Affiliated to Cancer Hospital, Chinese Academy of Medical Sciences/Cancer Hospital Affiliated to Shanxi Medical University, Taiyuan, China; ^2^ Department of Radiotherapy Oncology, Shanxi Province Cancer Hospital/Shanxi Hospital Affiliated to Cancer Hospital, Chinese Academy of Medical Sciences/Cancer Hospital Affiliated to Shanxi Medical University, Taiyuan, China; ^3^ Department of Pathology, Shanxi Province Cancer Hospital/Shanxi Hospital Affiliated to Cancer Hospital, Chinese Academy of Medical Sciences/Cancer Hospital Affiliated to Shanxi Medical University, Taiyuan, China; ^4^ Department of Imaging, Shanxi Province Cancer Hospital/Shanxi Hospital Affiliated to Cancer Hospital, Chinese Academy of Medical Sciences/Cancer Hospital Affiliated to Shanxi Medical University, Taiyuan, China

**Keywords:** perivascular epithelioid cell tumors, tyrosine kinase inhibitor, vascular endothelial growth factor receptor, anti-VEGFR, mammalian target of rapamycin inhibitor, malignant PEComa

## Abstract

Perivascular epithelioid cell tumors (PEComas) are rare mesenchymal tumors arising from perivascular epithelial cells. There was no standard treatment for unresectable PEComa before 2021. For a low incidence and a rarely curable disease, development of new therapy is essential. A 45-year-old female was diagnosed with malignant renal PEComa (likely with *TFE3* rearrangement) that underwent rapid progression after 10 months of surgery. The patient then received the tyrosine kinase inhibitor (TKI) Apatinib, and the tumor remained stable for 15 months before another progression. The patient then received the MTOR inhibitor everolimus that alleviated her symptoms but the tumor went into remission again after another 15 months. This result suggests that antagonizing the vascular endothelial growth factor receptor (VEGFR) pathway be a useful strategy for malignant PEComas, along with the MTOR pathway inhibition that had recently been approved for the rare tumor.

## Introduction

Perivascular epithelioid cell tumor (PEComa) is a tumor derived from mesenchymal tissue (1). In 2002, the World Health Organization (WHO) defined PEComas as mesenchymal tumors composed of perivascular epithelioid cells with unique histological and immunohistochemical features (2). The 2020 WHO classification of soft tissue tumors for PEComa includes renal angiomyolipoma (AML), pulmonary lymphangioleiomyomatosis (LAM), and PEComa-not otherwise specified (PEComa-NOS) (3). PEComa is more common in women and can occur in various organs, such as the pancreas, lungs, gastrointestinal tract, female reproductive system, abdominal cavity, pelvic cavity and retroperitoneum, urinary tract and skin. Other rare sites include nasal cavity, bone, oropharynx, and omentum (4–7). PEComas are usually treated by surgery (8, 9), and there was no standard treatment for cases with extensive metastases in the past. It was until 2021 that the MTOR inhibitor nab-sirolimus (Fyarro) was approved for advanced malignant PEComa, which remains to be the one and only therapeutic option approved for this rare disease to date (10). Here we report a case of PEComa originated from the right kidney. Extensive metastases in the abdomen and pelvis occurred 10 months after surgical ablation. The patient was then treated with the anti-angiogenic drug apatinib mesylate and her condition improved and remained stable for 15 months before a new progression. The patient was then treated with MTOR inhibitor everolimus and her condition was relieved again, however new progression was detected after another 15 months.

## Case report

**Figure 1:**
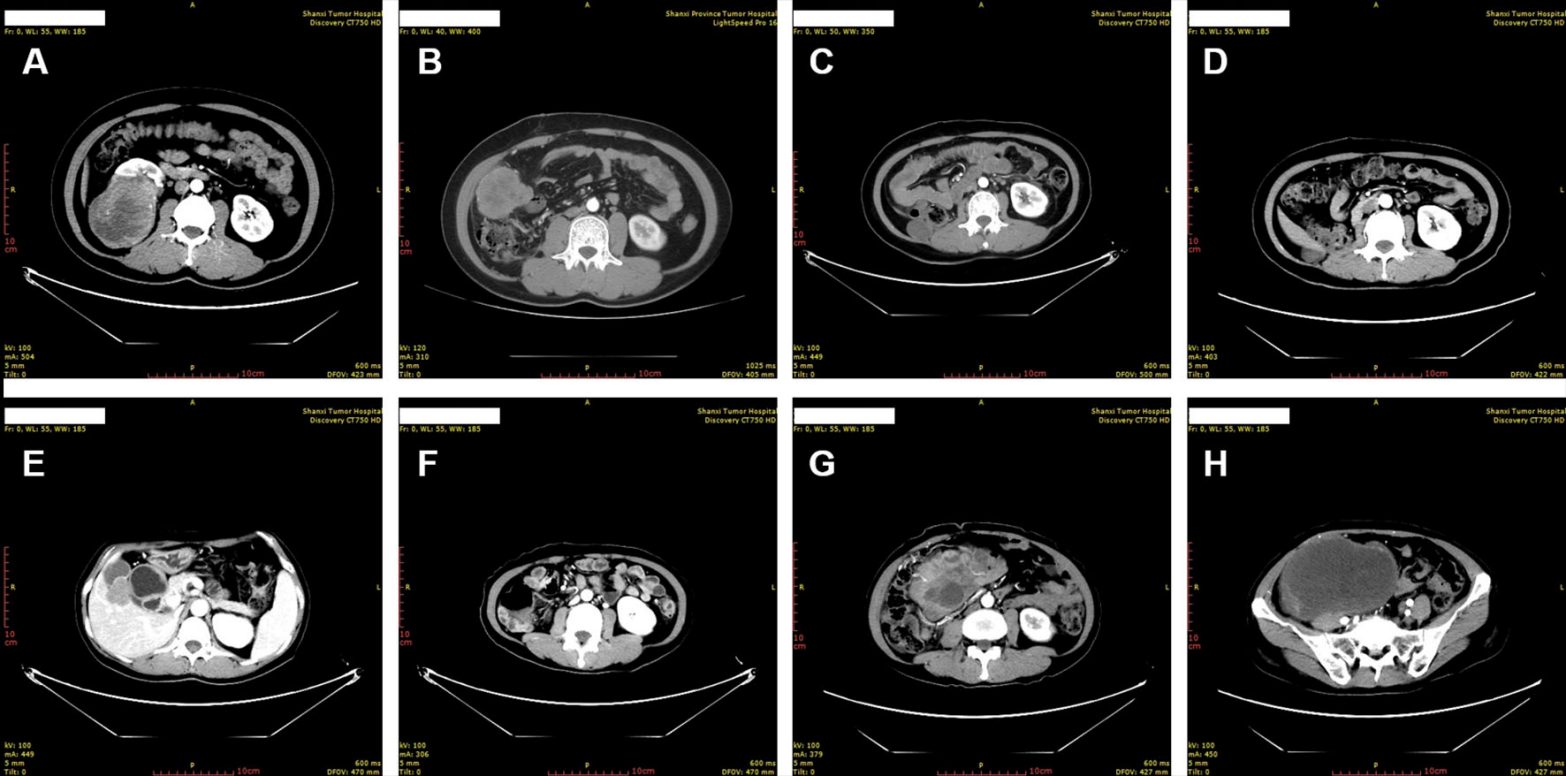
Chronological computed tomography of the perivascular epithelioid cell tumor. **(A)** The primary tumor was identified as right kidney mass. **(B)** Metastases were identified in the right abdominal cavity 10 months after resection of the primary tumor. **(C, D)** Shrinkage of the abdominal cavity tumor was identified 3 months **(C)** and 6 months **(D)** after the initiation of anti-VEGFR TKI treatment, respectively. **(E, F)** Continuous shrinkage of the abdominal cavity tumor and appearance of new metastases in gallbladder fossa was identified 15 months after the initiation of anti-VEGFR TKI treatment. **(G, H)** New, widespread metastases were identified in the abdomen and pelvis 15 months after the initiation of the MTOR inhibitor everolimus treatment.

**Figure 2:**
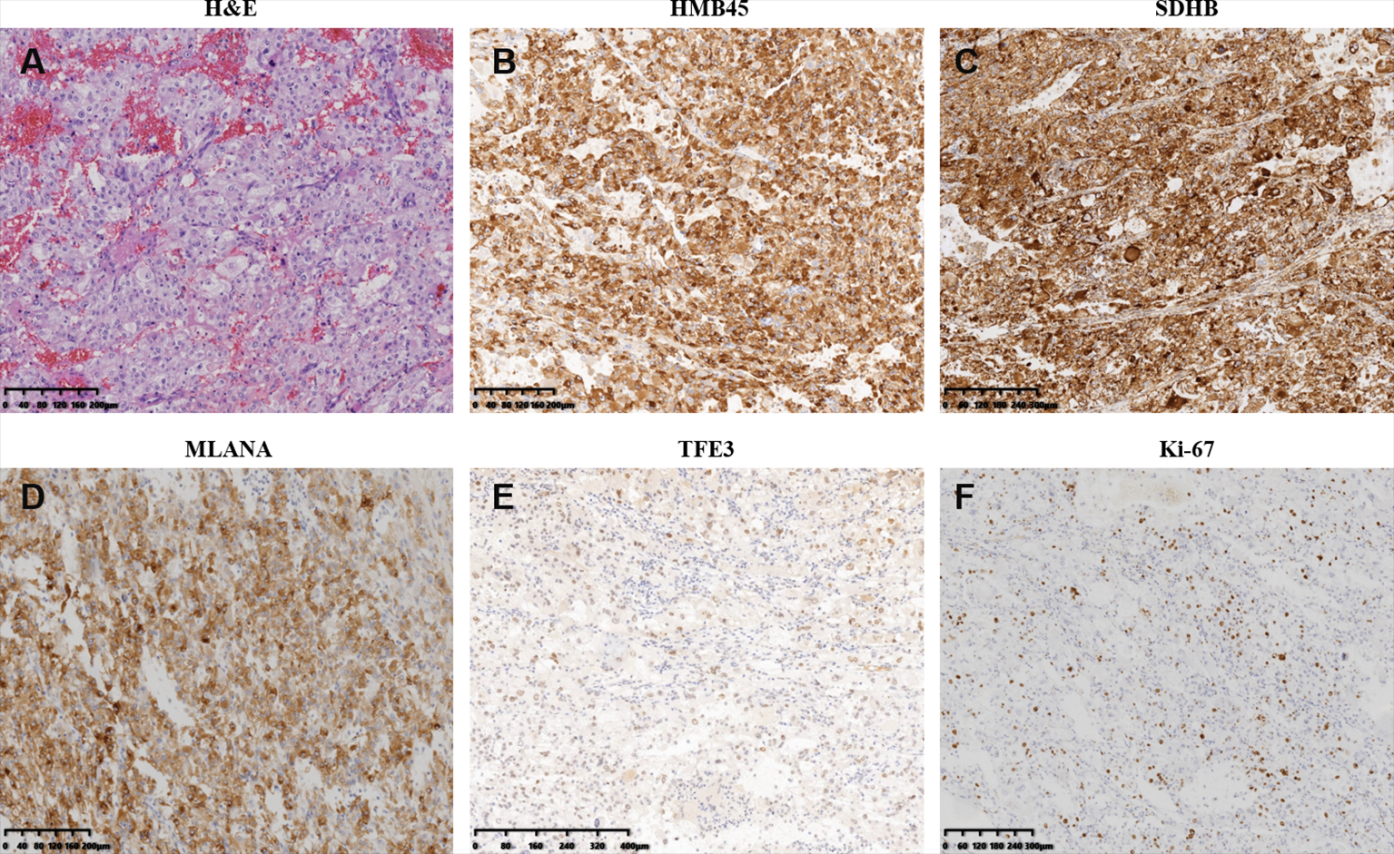
Pathological and immunohistochemistry features of the primary perivascular epithelioid cell tumor resected right kidney. **(A)** Hematoxylin–eosin staining of resection specimen. **(B–F)** Immunohistochemistry of select tumor markers: HMB45 **(B)**, SDHB **(C)**, MLANA **(D)**, TFE3 **(E)**, and Ki-67 **(F)**.

A 45-year-old woman without any complaint went to a local hospital for a wellness exam where a mass in her right kidney was detected by the abdominal ultrasonography. A second ultrasonography at Shanxi Provincial People’s Hospital showed that the size of the mass was about 6.2 × 5.7 cm. The computed tomography (CT) scan later performed in Shanxi Cancer Hospital showed right kidney mass of 8.2 × 7.1 cm indicative of kidney cancer (**Figure 1A**).Radical right nephrectomy was immediately performed under general anesthesia. The mass that was resected measured about 8 × 8 × 7 cm. In the operation, tumor thrombus in deep vein and vena cava were found and removed. There was no visible spreading in areas of the ureter, renal capsule, renal hilar blood vessels and other nerves and vessels. No enlarged lymph nodes were found beside the renal hilum and abdominal aorta. Pathology microscopy revealed atypical cells with large nucleus and abundant, pink-stained cytoplasm (**Figure 2A**). The nuclei appeared in vacuoles, with easily identifiable nucleoli showing mitotic characteristics (**Figure 2A**). Cells appeared in nests and sheets with abundant interstitial blood vessels, and were accompanied by necrosis in certain areas (**Figure 2A**). The tumor thrombus found in vena cava displayed similar histological abnormalities. Immunohistochemical stains showed tumor cells positive for KIT, P504S, SDHB, HMB45, MLANA, and TFE3 (weak positive), and negative for AE1/AE3, vimentin, MME, and CA9 (**Figures 2B–E** and data not shown). Ki-67 labeling index in the tumor cells was 15% (**Figure 2F**). The pathological findings supported the diagnosis of malignant PEComa.

**Figure 3:**
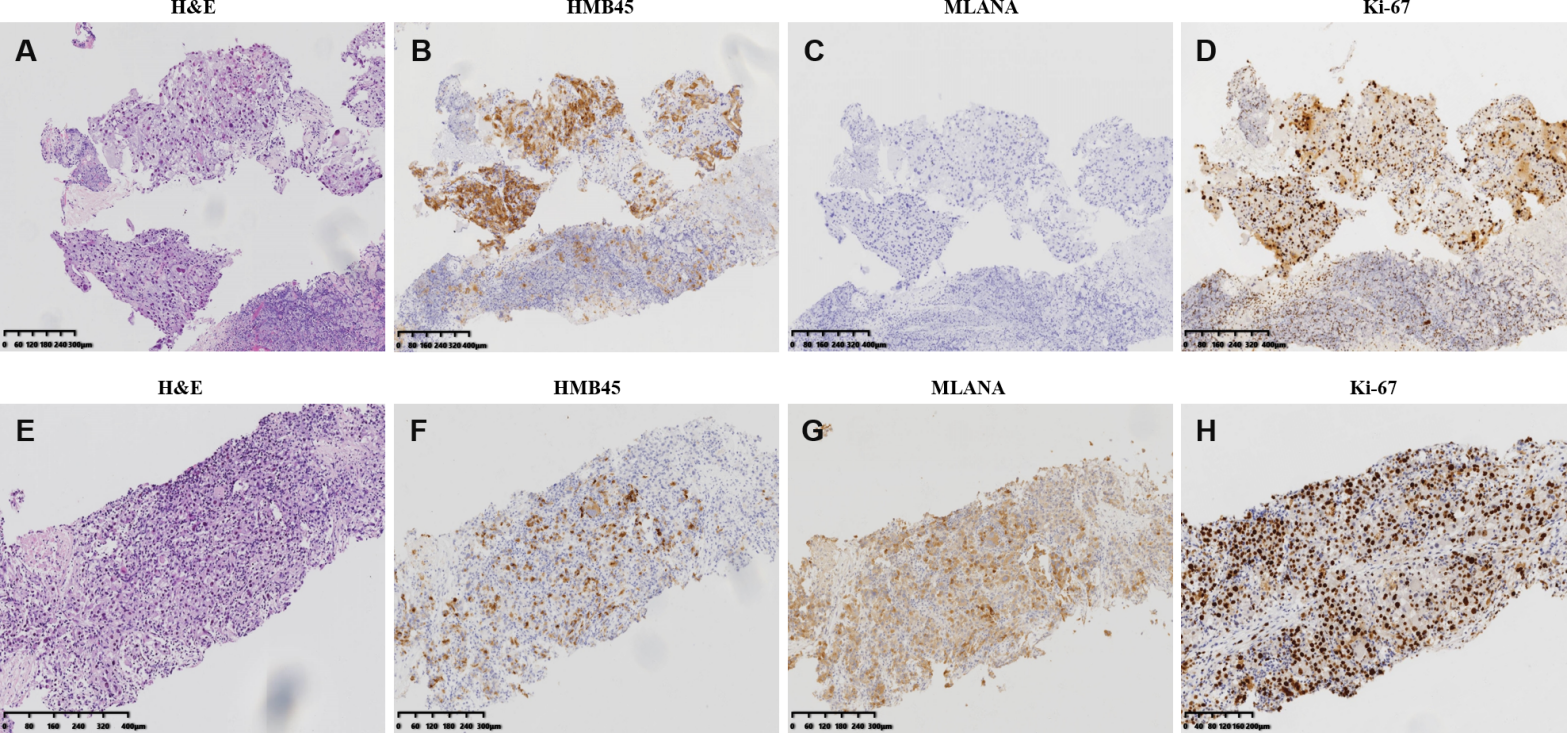
Pathological and immunohistochemistry features of the perivascular epithelioid cell tumor metastases. **(A–D)** hematoxylin–eosin staining **(A)** and imunohistochemistry of HMB45 **(B)**, MLANA **(C)**, and Ki-67 **(D)** of the biopsy specimen of the pelvis metastases identified 10 months after the resection of the primary tumor. **(E-H)** hematoxylin–eosin staining **(E)** and imunohistochemistry of HMB45 **(F)**, MLANA **(G)**, and Ki-67 **(H)** of the biopsy specimen of the gallbladder fossa metastases identified 15 months after pelvis manifestation and the initiation of anti-VEGFR TKI treatment.

**Figure 4:**
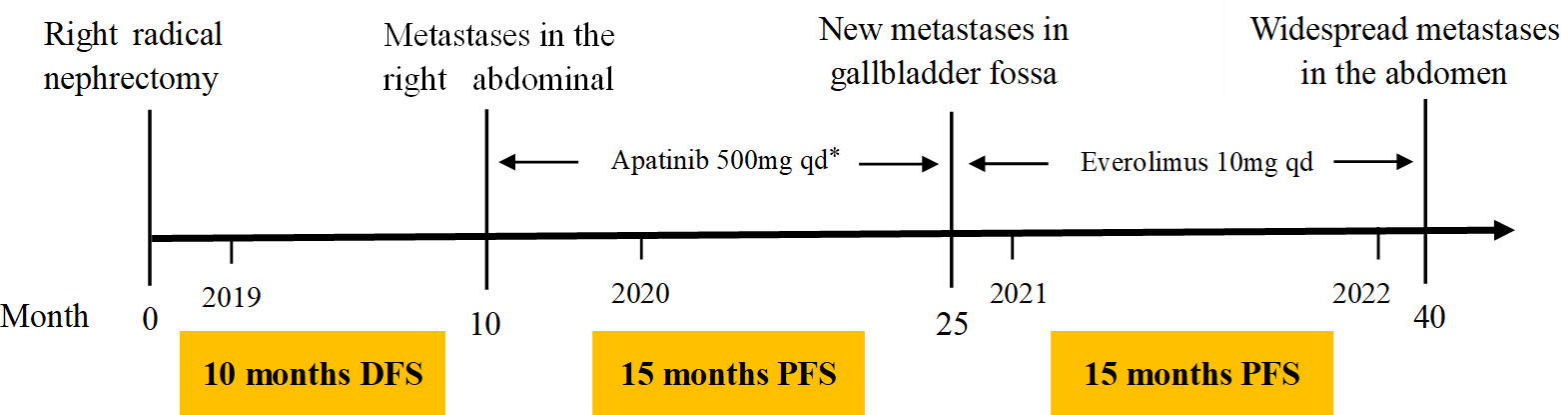
Patient treatment timeline. DFS, disease-free survival, PFS, progression-free survival. *Apatinib was started at 500 mg qd for 3 months and then increased to 750 mg qd for 10 days, but changed back to 500 mg qd due to the side effect at the high dosage (high blood pressure and nose bleeding).

The patient presented pain on the right lower back 10 months after the surgery. New CT scan revealed multiple nodules in the right abdominal cavity and lateral peritoneum and extraperitoneum, as well as the right side of the liver border, along with psoas muscle involvement and slightly enlarged retroperitoneal lymph nodes, indicative of metastasis (**Figure 1B**). A biopsy of the pelvic mass was immediately performed. The pathology showed that most of the tissues were necrotic, with a few degenerated cells bearing large nucleus (**Figure 3A**). Immunohistochemical stains showed tumor cells positive for HMB45 and TFE3, and negative for AE1/AE3, vimentin, MLANA, PAX8, S100, SMA (**Figures 3B, C** and data not shown). Ki-67 labeling index in the tumor cells was 40% (**Figure 3D**). The new pathological findings confirmed metastases of the primary PEComa. The patient was given the anti-VEGFR TKI apatinib mesylate about one week after the new diagnosis. The inhibitor was started at 500 mg qd for 3 months without apparent side effect. The dosage was then increased to 750 mg qd, however the patient’s blood pressure went up with occasional nose bleeding, so after 10 days the dosage was changed back to 500 mg qd. Overall the inhibitor appeared effective, leading to tumor shrinkage as revealed by follow-up CT scans (**Figures 1C, D**). However, 15 months later, the patient presented dull pain in the right upper quadrant accompanied with nausea but no vomiting. New CT scan showed a new gallbladder fossa mass, while previously identified nodules in the right abdominal cavity and lateral peritoneum and extraperitoneum became smaller (**Figures 1E, F**). An ultrasound-guided gallbladder fossa lymph node puncture was immediately performed. The pathology identified irregular cells with deviated nuclei (**Figure 3E**). Immunohistochemical stains showed tumor cells positive for HMB45, MLANA, vimentin, KIT and S100 (partially), and negative for AE1/AE3, MME and calponin (**Figures 3F, G** and data not shown). Ki-67 labeling index in the tumor cells was 80% (**Figure 3H**). The new pathological findings confirmed new metastases. The patient was given the MTOR inhibitor everolimus mesylate (10 mg qd) that gradually alleviated her symptoms (nausea and abdominal pain). The only side effect she experienced was mild fatigue. However, the follow-up CT scan after another 15 months manifested progression with extensive metastases in the abdomen and pelvis (**Figures 1G, H**). The patient refused new treatment and is currently living with the tumor. The disease progression and the responses to the evolving treatment is summarized in **Figure 4**.

## Discussion

PEComas are rare tumors with extremely low incidence. Most of the published studies on PEComa have been case reports or retrospective analysis of small samples (11–13). The symptoms of PEComa are not specific and largely depend on the location of the primary tumor. It usually presents as a painless mass which lacks specific imaging findings, and the diagnosis relies on pathological and immunohistochemical features. Histopathologically, the tumor is mainly composed of epithelioid and spindle cells with vast blood vessels in the stroma, and the tumor cells often reside radially or sheath-like around the expanding blood vessels (14, 15). Immunohistochemically, tumor cells are also stained positive for melanocyte markers such as HMB45 and MLANA, and the smooth muscle actin (SMA). Some PEComas are also positive for CD31, CD34, KIT and S100 (16, 17), In addition, diffuse and strong nuclear expression of TFE3 in PEComa often suggests *TFE3* gene translocation. In this case report, the primary kidney tumor and subsequent metastases from pelvis (found 10 months after the surgery) and gallbladder fossa (found 15 months after starting the apatinib mesylate treatment) all stained positive for HMB45. The primary tumor and gallbladder fossa metastases also stained positive for MLANA, with the latter also positive for S100. These immunohistochemical findings supported the diagnosis of PEComa.

Most PEComas tend to be benign in their biological behavior, and only a few are invasive or malignant. In 2005, Folpe et al. (18) proposed the diagnostic criteria for malignant PEComa: tumour size > 5 cm, infiltration of surrounding tissues, increased cytological and nuclear atypia, high mitotic activity (mitotic figures ≥1/50 high-power field), presence of necrosis and vascular invasion. Malignancy is considered when there are ≥2 indications; indeterminate of malignant potential is considered when the tumor diameter is > 5 cm or the tumor cells present pleomorphic nuclear morphology or appear as multinucleated giant cells; benign tumors are considered to those without the above malignant indications. The 2020 WHO classification of soft tissue tumors classifies PEComas into benign and malignant ones, and states that malignant PEComas are usually larger in size, exhibit pronounced nuclear atypia and pleomorphism, distinct mitotic figures and infiltrative borders, and tend to be aggressive in clinical course. This patient is in line with the pathological manifestations of malignant PEComa. Notably, with the progression of the disease the expression of Ki-67 in tumor cells gradually increased, indicating that the tumor malignancy might also increase along with enhanced invasiveness. It also suggests that new operation or biopsy should be performed in a timely manner to obtain the latest pathological diagnosis for relapsed or metastatic PEComa, as the tumor’s biological behavior may have changed.

Surgery is currently considered the preferred method for the treatment of PEComa, and most patients can achieve good prognosis after surgery (19, 20). There has been a lack of effective treatment for locally advanced unresectable or extensively metastatic malignant PEComa as conventional chemotherapy or radiotherapy has no obvious effect (21). Indeed, although previous studies have shown (22, 23) that gemcitabine- or anthracycline-based chemotherapies could be an option for the treatment of malignant PEComa, they are not widely used as they may be effective only for a small number of patients and the progression-free survival after treatment is usually short. Anti-angiogenic agents are important therapeutic drugs for vascularized soft tissue tumors. PEComas are rich in abnormal blood vessels, and anti-angiogenic therapy should theoretically be effective (21, 24). In line with this, Radzikowska Edeng et al. found that serum VEGF-D is a useful biomarker of lymphangioleiomyomatosis (LAM, one special type of PEComa) that correlates with disease severity and might also prove predictive towards therapeutic decision (25). Further study showed that the VEGFR inhibitor axitinib attenuated the VEGF-D levels in the serum and lung lining, and more importantly, reduced Tsc2-null lung lesion growth in a mouse model of LAM (26). The small molecule inhibitor apatinib mesylate functions as an anti-angiogenic TKI by highly selective inhibition of VEGFR-2 (27). It had been approved in China for third-line treatment of advanced gastric cancer (28), and also showed efficacy in esophageal cancer (29), liver cancer (30) and soft tissue sarcoma (31). In August 2019, we administered apatinib mesylate to this patient, which led to progression-free survival (PFS) of 15 months. In another case study published in 2020, apatinib was administered for the treatment of gastrointestinal PEComa with *TFE3* rearrangement that resulted in PFS for 7 months (32). More recently, Liapi et al. reported a case of uterine metastatic PEComa that progressed on MTOR inhibitor, while subsequent treatment with the VEGFR inhibitor pazopanib led to regression and stability of multiple metastases (33). Taken together, our report along with other studies suggest that the anti-angiogenic TKIs could be effective for the treatment of some malignant PEComas. Recent studies had also identified mutations or chromosome translocations in sporadic PEComa cases that may promote tumorigenesis through activation of the MTOR signaling pathway, providing rationales for MTOR inhibitors (such as rapamycin, sirolimus, and everolimus) as a new therapeutic option for PEComas (34–36). As reported at the American Society of Clinical Oncology meeting in 2020 (37), Fyarro (sirolimus albumin-bound particles) monotherapy of PEComas achieved an overall response rate of 39% (95% confidence interval (CI): 22% - 58%) with one complete responder and 11 partial responders. In addition, 52% of patients had stable disease. The PFS was 10.6 months (95% CI:5.5 months - not reached), and the median overall survival was 40.8 months (95% CI:22.2 months - not reached). Based on this, the United States Food and Drug Administration (FDA) approved Fyarro for the treatment of adult patients with locally advanced unresectable or metastatic malignant PEComas. It is the first and only FDA-approved drug for the treatment of advanced malignant PEComa, yet not available in China. Instead, we gave the patient another MTOR inhibitor everolimus after the progression on apatinib, which was effective to relieve her symptoms. However, after 15 months, the follow-up CT scan manifested new progression. Nevertheless, combination of MTOR inhibitors with VEGFR TKIs might have synergistic effect for the treatment of refractory PEComa (38). In addition, further elaboration of the molecular mechanism of PEComa tumorigenesis may be beneficial to the exploit of new treatments. It has been found that *TSC* gene mutation and *TFE3* gene rearrangement lead to two different pathways in the occurrence of PEComa (39, 40). *TFE3*-rearranged PEComa lacks *TSC1/2* gene mutation, which is associated with MTOR pathway activation. Some cases are insensitive or unresponsive to MTOR inhibitors, and there may be *TFE3* gene translocation. As mentioned above, antiangiogenic drug may be an option for these patients, and targeted drugs for tumors associated with *TFE3* gene rearrangement are also emerging. It had been shown in a recent trial that the MET TKI achieved clinical responses in *TFE3*-rearranged acinar soft tissue sarcoma (41). Such novel drugs may become new options for the treatment of PEComas with *TFE3* translocation. With regard to this patient, we did show TFE3 positive staining of the primary tumor, which implies plausible *TFE3* gene rearrangement, however neither *TFE3* rearrangement nor the mutation status of the *TSC1/2* genes was confirmed by genetic testing due to the patient’s refusal. The patient also refused new treatment and future follow-up. Future studies with genetic testing and long-term follow-ups are warranted.

## Conclusion

PEComas are rare tumors with considerable heterogeneity in their biological behaviors. Surgery remains the best treatment for resectable PEComa. For unresectable malignant PEComa, MTOR inhibitors have become the standard treatment. In the case reported here, new lesions were developed after resection, but the patient achieved remission with the antiangiogenic TKI, suggesting that such drugs are also beneficial in the treatment of malignant PEComa. Directions of future studies will include the timing of antiangiogenic drug administration, potential combination with MTOR inhibitors, and new treatment options for MTOR inhibitor resistant PEComas.

## Data availability statement

The original contributions presented in the study are included in the article/supplementary material. Further inquiries can be directed to the corresponding author.

## Ethics statement

The studies involving human participants were reviewed and approved by Shanxi Province Cancer Hospital Ethics Committee. The patients/participants provided their written informed consent to participate in this study.

## Author contributions

NZ, YR, JZ, LW, and YW collected clinical data. LZ contributed to the collection of pathological data. XZ contributed to the collection of tomographic data. NZ, YR, and YW conducted the literature review and wrote the manuscript. NZ and YR contributed equally to the manuscript. YW oversaw the study. All authors contributed to the article and approved the submitted version.

## Conflict of interest

The authors declare that the research was conducted in the absence of any commercial or financial relationships that could be construed as a potential conflict of interest.

## Publisher’s note

All claims expressed in this article are solely those of the authors and do not necessarily represent those of their affiliated organizations, or those of the publisher, the editors and the reviewers. Any product that may be evaluated in this article, or claim that may be made by its manufacturer, is not guaranteed or endorsed by the publisher.
